# PRECORDIAL PAIN, LEUKOCYTOSIS AND BICYTOPENIA IN A TEENAGER WITH SYSTEMIC JUVENILE IDIOPATHIC ARTHRITIS UNDER IMMUNOSUPPRESSIVE THERAPY

**DOI:** 10.1590/1984-0462/;2019;37;2;00004

**Published:** 2019-02-25

**Authors:** Marina de Sousa Vieira, Flávia Regina de Andrade, Letícia Maria Kolachinski Raposo Brandão, Virgínia Tafas da Nóbrega, Vanessa Ramos Guissa, José Roberto Provenza

**Affiliations:** a Pontifícia Universidade Católica de Campinas, Campinas, SP, Brazil.

**Keywords:** Juvenile idiopathic arthritis, Pericarditis, Myocarditis, Macrophage activation syndrome, Immunosuppression, Artrite juvenil idiopática, Pericardite, Miocardite, Síndrome de ativação macrofágica, Imunossupressão

## Abstract

**Objective:**

To highlight the importance of the new classification criteria for the macrophage activation syndrome (MAS) in systemic juvenile idiopathic arthritis in order to reduce morbidity and mortality outcome related to this disease.

**Case description:**

A 12-year-old female patient with diagnosis of systemic juvenile idiopathic arthritis under immunosuppression therapy for two years developed cough, acute precordial chest pain, tachypnea, tachycardia and hypoxemia for two days. Chest tomography showed bilateral laminar pleural effusion with bibasilar consolidation. The electrocardiogram was consistent with acute pericarditis and the echocardiogram showed no abnormalities. Laboratory exams revealed anemia, leukocytosis and increased erythrocyte sedimentation rate, as well as C-reactive protein rate and serum biomarkers indicative of myocardial injury. Systemic infection and/or active systemic juvenile idiopathic arthritis were considered. She was treated with antibiotics and glucocorticoids. However, 10 days later she developed active systemic disease (fever, evanescent rash and myopericarditis with signs of heart failure) associated with macrophage activation syndrome, according to the *2016 Classification Criteria for Macrophage Activation Syndrome in Systemic Juvenile Idiopathic Arthritis*. She was treated for five days with pulse therapy, using glucocorticoids, immunoglobulin and cyclosporine A, with improvement of all clinical signs and laboratory tests.

**Comments:**

Myopericarditis with signs of heart failure associated with MAS is a rare clinical presentation of systemic juvenile idiopathic arthritis. Macrophage activation syndrome occurs mainly during periods of active systemic juvenile idiopathic arthritis and may be triggered by infection. Knowledge about this syndrome is crucial to reduce morbidity and mortality.

## RESUMO

**Objetivo:**

Destacar a importância do conhecimento sobre os novos critérios de classificação para síndrome de ativação macrofágica (SAM) na artrite idiopática juvenil sistêmica para reduzir a morbidade e mortalidade desse desfecho.

**Descrição do caso:**

Adolescente do sexo feminino de 12 anos de idade, em terapia imunossupressora por diagnóstico de artrite idiopática juvenil sistêmica há 2 anos, com quadro de tosse, dor precordial aguda, taquipneia, taquicardia e hipoxemia há 2 dias. A tomografia de tórax evidenciou efusão pleural laminar bilateral com consolidação bibasal. O eletrocardiograma foi compatível com pericardite aguda, e o ecocardiograma foi normal. Os exames laboratoriais revelaram anemia, leucocitose e aumento da velocidade de hemossedimentação, proteína C-reativa e marcadores séricos de lesão miocárdica. Infecção sistêmica e/ou doença sistêmica em atividade foram consideradas. A paciente foi tratada com antibióticos e glicocorticoide. Entretanto, dez dias depois, evoluiu com doença sistêmica em atividade (febre, exantema e miopericardite com insuficiência cardíaca) associada à SAM, de acordo com o *2016 Classification Criteria for Macrophage Activation Syndrome in Systemic Juvenile Idiopathic Arthritis,* e necessitou de cinco dias de pulsoterapia com glicocorticoide, imunoglobulina e ciclosporina A, com melhora de todos os parâmetros clínicos e laboratoriais.

**Comentários:**

A miopericardite com sinais de insuficiência cardíaca associada à SAM é uma apresentação clínica rara da artrite idiopática juvenil sistêmica, que ocorre principalmente em períodos de atividade sistêmica da doença e pode ser deflagrada por infecções. O conhecimento sobre essa síndrome é fundamental para reduzir morbidade e mortalidade desse grave desfecho.

## INTRODUCTION

Systemic juvenile idiopathic arthritis (sJIA) accounts for only 10-20% of all chronic childhood arthritis cases. At onset, cases may mimic infection, malignancy, inflammatory bowel disease, vasculitis, inflammatory and other autoimmune diseases.^1^ ^,^ ^2^ ^,^ ^3^ According to the International League of Associations for Rheumatology (ILAR) classification criteria for juvenile idiopathic arthritis,^4^ sJIA in youngsters under 16 years of age with arthritis includes patients with at least two weeks of fever associated with one or more of the four following conditions: evanescent rash, generalized lymphadenopathy, liver or spleen enlargement and serositis. Infections, amyloidosis, cardiac or pulmonary activity and macrophage activation syndrome (MAS) can be potentially the cause of fatal complications.^3^ MAS is an intense systemic inflammatory condition mediated by continuous activation and expansion of T lymphocytes and macrophages count, resulting in hypersecretion of proinflammatory cytokines. MAS occurs in approximately 10% of patients with systemic active sJIA and may be triggered by infection. Mortality rate has been reported to be 8%.^1^

Between April 2014 and June 2017, 270 pediatric patients were monitored at the Pediatric Rheumatology Unit of Pontifícia Universidade Católica de Campinas. Among those patients, 48 were diagnosed with juvenile idiopathic arthritis (JIA); three patients exhibited the subtype sJIA (6.3%) among which one presented myopericarditis with signs of heart failure associated with MAS. This case is described hereafter. This case report has been approved by the Local Ethics Committee of the University Hospital.

## CASE DESCRIPTION

A 12-year-old female patient was admitted at the University Hospital of Pontifícia Universidade Católica de Campinas, São Paulo, reporting a 2-day history of cough and acute precordial chest pain, without fever or other systemic symptoms. Her past medical history revealed sJIA (daily fever, macular rash and polyarthritis) diagnosed two years before, and she had been monitored in the Pediatric Rheumatology Unit of the University Hospital during the past 16 months, without systemic manifestations since the first month of diagnosis. Active arthritis and serum inflammatory biomarkers had not been detected during the last five months of follow up. She had been under immunosuppressive therapy with prednisone (24 months), leflunomide (16 months) and etanercept (five months).

On admission to the emergency room, the patient exhibited normal state of consciousness, tachypnea (30 breaths/minute), tachycardia (141 beats/minute), 88% peripheral oxygen saturation on room air, blood pressure 120/60 mmHg, normal cardiac and lungs auscultation. There was no articular activity. The chest x-ray showed small bibasilar lung consolidation. Chest tomography evidenced bilateral laminar pleural effusion with bibasilar consolidation and atelectasis. Screening for active and latent tuberculosis infection was negative. Vaccination was up to date according to patient’s age, including recent influenza immunization. Laboratory exams revealed hemoglobin 10.5 g/dL, hematocrit 34.2%, erythrocyte mean corpuscular volume (MCV) 77, white blood cell count 23.290/mm^3^ (24% band neutrophils, 50% neutrophils, 23% lymphocytes, 0% eosinophils and 3% monocytes), platelets 307.000/mm^3^, erythrocyte sedimentation rate (ESR) 99 mm/1^st^ hour (normal <11), C-reactive protein (CRP) 48 mg/L (normal<0.5), normal urinalysis, urea 21 mg/dL (normal 16.6-48.5), creatinine 0.28 mg/dL (normal 0.53-0.79), aspartate aminotransferase (AST) 54 IU/L (normal 0-32), alanine aminotransferase (ALT) 19 IU/L (normal 0-33), creatine kinase (CK) 110 U/L (normal <170), creatine phosphokinase MB (CK-MB) 36 U/L (normal 7-25) and troponin 0.447 ng/mL (normal <0.013). Bacterial culture screening was negative. Electrocardiogram was consistent with acute pericarditis (widespread ST segment elevation). The echocardiogram demonstrated normal ventricular function, without pericardial effusion. Systemic infection and/or active sJIA were considered. The patient was treated with antibiotics (piperacillin/tazobactam and teicoplanin) and glucocorticoids (2 mg/kg/day). Immunosuppression was discontinued. Serologic tests for viral myocarditis were not performed.

**Figure 1:**
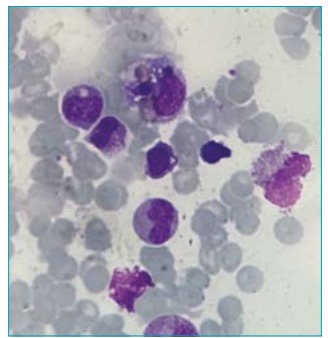
Hemophagocytic activity in bone marrow aspirate.

Vital signs were stable and within normal limits 72 hours after support. However, 10 days later the patient developed fever (38.6-38.8ºC), evanescent rash and tachycardia. At the time, she was still active, without other abnormalities on physical exam. Cardiovascular magnetic resonance showed signs of acute myopericarditis (absence of myocardial edema, presence of myocardial fibrosis in the basal inferolateral wall with subepicardial pattern and in the pericardium near the right ventricular wall). Chest radiograph was normal. Exams revealed hemoglobin 10 g/dL, leukocytosis (29.330/mm^3^ leukocytes), drop in platelets count (111.000/mm^3^) and increased ESR to 66 mm/1^st^ hour, CRP 24 mg/dL, AST 231 IU/L, triglycerides 190 mg/dL (normal <90), ferritin >2.000 ng/mL (normal 13-150) and INR 2.75 (normal <1.25). Diagnosis of active sJIA was established. MAS was suspected. The main differential diagnosis was sepsis. The patient received cefepime and methylprednisolone pulse therapy (30 mg/kg/day). After the second infusion of glucocorticoids, the patient complained again of precordial pain and presented hypotension, bradycardia and dyspnea. At this time, the echocardiogram demonstrated reduced left ventricular ejection function (<45%), mild mitral insufficiency, moderate tricuspid insufficiency and mild pericardial effusion. The patient’s exams showed abrupt drop in hemoglobin 6.5 g/dL, platelets 14.000/mm^3^, ESR 7 mm/1^st^ hour, CRP 1.98 mg/L and fibrinogen 61 mg/dL (normal 200-400) and high AST level (126 IU/L), triglycerides 314 mg/dL, ferritin >2.000 ng/mL and INR 1.36. The serum markers of myocardial injury were positive (CK-MB 121 U/L and troponin 0.139 ng/mL). Bone marrow aspirate showed mild hemophagocytosis (Figure 1). Diagnosis of MAS was established according to the new MAS criteria in sJIA patients.^1^ The patient was treated once more with intravenous methylprednisolone (30 mg/kg/day) for five days, oral cyclosporine A (5 mg/kg/day) and intravenous immunoglobulin (2 g/kg) with improvement of all clinical and laboratory standards. Twenty-seven days after admission, the patient received the first dose of tocilizumab, associated with prednisone and cyclosporine A, which caused a prompt decrease in the disease activity (Table 1).

**Table 1:** Clinical features, laboratory exams and therapeutic follow-up of a patient with systemic juvenile idiopathic arthritis and macrophage activation syndrome.

	Day 1	Day 10	Day 12	Day 27	Day 60
Clinical features					
Arthritis	-	-	-	-	-
Fever	-	+	-	-	-
Rash	-	+	-	-	-
Lymphadenopathy	-	-	-	-	-
Laboratory exams					
Hemoglobin, g/dL	10.5	10.0	6.5	10.7	13.8
Platelets count, /mm^3^	307,000	111,000	14,000	234,000	212,000
White blood cell, /mm^3^	23,290	29,330	11,340	12,940	15,200
CRP, mg/L	48	24	1.98	13.36	<0.5
ESR, mm/1^st^ hour	99	66	7	113	2
AST, IU/L	54	231	126	46	22
Fibrinogen, mg/dL		243	61	227	
Ferritin, ng/mL		>2000	>2000	>2000	
Triglycerides, mg/dL		190	314	146	62
Therapy					
Glucocorticoids, mg/kg/d	0.4	2	30 (D2/5)	2	2
Cyclosporine A, mg/kg/d	-	-	5	5	5
Immunoglobulin, g/kg/d	-	-	2	-	-
Etanercept, mg/kg	0.8 (1x/wk)	-	-	-	-
Leflunomide, mg/d	20	-	-	-	-
Tocilizumab, mg/kg	-	-	-	8 (every 2 weeks)	8 (every 2 weeks)

CRP: C-reactive protein; ESR: erythrocyte sedimentation rate; AST: aspartate aminotransferase; mg/kg/d: mg/kg/day; g/kg/d: g/kg/day; 1x/wk: once a week.

## DISCUSSION

The treatment used in juvenile rheumatic patients may induce immunosuppression with consequent enhancement of infection susceptibility, mainly due to the use of steroids or tumor necrosis factor alpha (TNF-alpha) antagonists.^5^ ^,^ ^6^ Pneumonia should be considered at the first signs of acute respiratory failure, as was observed in the reported patient. In sJIA, pleuritic chest pain is observed with severe effusion and progressive dyspnea. Pleural effusion is the most common asymptomatic respiratory manifestation, generally detected only as an incidental finding in chest radiographs and it may occur in association with pericarditis. Parenchymal pulmonary diseases, such as pulmonary fibrosis, alveolar proteinosis or lipoid pneumonia, are rare and in general unresponsive to multiple medications.^3^ ^,^ ^7^

Myopericarditis diagnosis is based on precordial chest pain. Positive myocardial injury markers, electrocardiogram, echocardiogram and magnetic resonance were the first indicators of systemic activity flare in this patient. Chest pain, with or without dyspnea, is a common symptom of acute pericarditis in sJIA, and reports indicate that 81% of children exhibit echocardiogram abnormalities during periods of systemic activity; however, the absence of pericardial effusion does not exclude pericarditis. Typical chest pain associated with widespread ST segment elevation in the electrocardiogram confirm the diagnosis of inflammatory pericardial syndrome, according to the *2015 European Society of Cardiology guidelines on pericardial diseases*.^3^ ^,^ ^8^ ^,^ ^9^ ^,^ ^10^ Myocarditis is a rare clinical presentation of sJIA and may result in heart failure.^3^ ^,^ ^9^

Laboratory in sJIA indicates a highly inflammatory disease, with leukocytosis (often leukocytes above 30.0000 cells/mm^3^) and thrombocytosis. Anemia is common, with hemoglobin in the range of 7-10g/dL (hypochromic); however, anemia can also be normocytic or microcytic. The ESR, CRP, ferritin, fibrinogen and d-dimer levels are often elevated. In contrast, patients with active systemic disease who experienced abrupt drop in leukocytes, platelet counts, ESR and fibrinogen associated with high level of liver enzymes, lactate dehydrogenase (LDH) and triglycerides, as in this case, must be promptly recognized and investigated for MAS.^3^ ^,^ ^11^ ^,^ ^12^ ^,^ ^13^

It is reported that subclinical MAS may occur in up to 40% of patients with sJIA.^1^ ^,^ ^3^ ^,^ ^14^ MAS clinical heterogeneity may mimic systemic JIA or systemic infections flares.^1^ Although MAS and hemophagocytic lymphohistiocytosis (HLH) are physiopathologically and clinically similar, diagnosis of MAS with the use of HLH diagnostic guidelines^15^ has several limitations in sJIA patients. Due to the inflammatory status of this rheumatic disease, leucopenia may not occur and the typical clinical symptoms (with the exception of fever) observed in HLH are often delayed and/or may mimic other inflammations or systemic infections.^1^ The typical MAS histopathological feature (hemophagocytic activity in bone marrow biopsy specimens or aspirate) may not be present in the initial stages and lacks specificity for HLH.^1^ ^,^ ^15^ According to recent validated criteria by the European League Against Rheumatism^1^ and the American College of Rheumatology,^1^ diagnosis of MAS is established if a patient with sJIA or suspected sJIA presents with fever associated with ferritin >684 ng/mL and any of the following four criteria: platelet count ≤181.000/mm^3^, AST >48 IU/L, triglycerides >156 mg/dL, fibrinogen ≤360 mg/dL. These new criteria provide 0.73 sensitivity and 0.99 specificity for MAS classification in sJIA patients. Considering the clinical evolution of the reported case, the patient fulfilled the new criteria for MAS in sJIA from the onset of the period of fever, including the drop in the platelets count associated with high levels of AST, triglycerides and ferritin. The mild macrophage activity in bone marrow aspirate can be explained because the aspirate was performed after two cycles of pulse steroid therapy, which decreases macrophage activity or because of the earlier glucocorticoid high-dose therapy when the first signs of incipient MAS were suspected and observed.

In conclusion, MAS occurs mainly during periods of active sJIA and may be triggered by infections as was the case reported herein. Awareness of the 2016 MAS classification criteria in sJIA is crucial to reduce this disease morbidity and mortality outcome, because the clinical, laboratorial and histopathological findings may not be HLH specific. Myopericarditis with signs of heart failure associated with MAS is rare and justifies five days of pulse therapy with glucocorticoids, immunoglobulin and cyclosporine A.
